# Comparative Study of Eleven Mechanical Pretreatment Protocols for *Cryptosporidium parvum* DNA Extraction from Stool Samples

**DOI:** 10.3390/microorganisms9020297

**Published:** 2021-02-02

**Authors:** Laure Claudel, Nicolas Valeix, Louise Basmaciyan, Bruno Pereira, Damien Costa, Anne Vincent, Stéphane Valot, Loic Favennec, Frederic Dalle

**Affiliations:** 1 Laboratoire de Parasitologie-Mycologie, Plateforme de Biologie Hospitalo-Universitaire, 2 rue A. Ducoudray, BP 37013, CEDEX, 21070 Dijon, France; laure.claudel@chu-dijon.fr (L.C.); nicolas.valeix@chu-dijon.fr (N.V.); louise.basmaciyan@chu-dijon.fr (L.B.); anne.lamy@chu-dijon.fr (A.V.); stephane.valot@chu-dijon.fr (S.V.); 2 UMR PAM Univ Bourgogne Franche-Comté-AgroSup Dijon-Equipe Vin, Aliment, Microbiologie, Stress, CEDEX, 21078 Dijon, France; 3 Unité de Biostatistiques, Direction de la Recherche Clinique (DRCI), CHU de Clermont-Ferrand, 63000 Clermont-Ferrand, France; bpereira@chu-clermontferrand.fr (B.P.); loic.favennec@chu-rouen.fr (L.F.); 4 Laboratoire de Parasitologie-Mycologie, Centre Hospitalo-Universitaire C. Nicolle de Rouen, 76000 Rouen, France; damien.costa@chu-rouen.fr; 5 Centre National de Référence–Laboratoire Expert des Cryptosporidioses, Institut de Biologie Clinique, Centre Hospitalo-Universitaire C. Nicolle de Rouen, 76000 Rouen, France

**Keywords:** *Cryptosporidium parvum*, mechanical pretreatment, DNA extraction, stool samples, real-time PCR, molecular diagnosis

## Abstract

Nowadays, many commercial kits allow the polymerase chain reaction (PCR) detection of *Cryptosporidium* deoxyribonucleic acid (DNA) in stool samples, the efficiency of which relies on the extraction method used. Mechanical pretreatment of the stools using grinding beads has been reported to greatly improve this extraction step. However, optimization of this key step remains to be carried out. Indeed, many parameters could influence the pretreatment performances, among which the modulation of the speed and duration of the grinding step, in addition to the physicochemical features of the grinding beads, have never been evaluated to date. In this study, eleven commercial mechanical pretreatment matrixes (Lysis matrix tubes^®^, MP Biomedical, Irvine, CA, USA) composed of beads with different sizes, shapes, and molecular compositions, were evaluated for their performances in improving *Cryptosporidium parvum* oocyst DNA extraction before amplification by using our routinely used real-time PCR method. As expected, the eleven commercial mechanical pretreatment matrixes showed varying performances depending on the composition, size, and shape. All in all, the best performances were obtained when using the Lysing matrix, including ceramic beads with a median size (diameter of 1.4 mm).

## 1. Introduction

*Cryptosporidium* sp. is a protozoan parasite of medical and veterinary importance that causes gastroenteritis in a variety of vertebrate hosts, including humans. Transmission occurs through the fecal–oral route, by the ingestion of viable oocysts excreted in the environment by infected hosts. Because of their directly contaminating feature, ingestion of drinking water or food contaminated by *Cryptosporidium* oocysts can lead to epidemics affecting numerous people. In healthy subjects, cryptosporidiosis is asymptomatic or expresses as a diarrhea most often self-resolving. However, severe and prolonged infections are observed in immunocompromised patients and children under five [1,2]. The most frequent species isolated in humans are *C. parvum* and *C. hominis*, encompassing more than 90% of the cases of human cryptosporidiosis diagnosed in France [3]. Because treatment options remain limited, infection prevention and control measures are critical for the protection of vulnerable populations [4]. Thus, efficient diagnostic tools are needed for *Cryptosporidium* sp. detection in stool specimens from patients presenting with severe infection, as well as from asymptomatic carriers.

Nowadays, molecular diagnosis methods for the detection of *Cryptosporidium* sp. in stool samples are increasingly replacing microscopic techniques, and are sensitive, specific, and less time-consuming [5,6,7,8]. Molecular methods are multi-step procedures (including a pretreatment step, DNA extraction and amplification, detection of amplified PCR products. and data analysis), each step influenced by various parameters that need to be properly optimized. Some studies have already evaluated the performances of manual and automated systems for *Cryptosporidium* sp. DNA extraction from oocysts in stools [9,10,11,12,13,14]. However, the *Cryptosporidium* oocyst wall is a robust thick structure, composed of three distinct layers that combine to protect the internal sporozoïtes, and is also responsible for difficulties in extracting DNA by conventional methods [15,16,17]. Consequently, a pretreatment step aimed at disrupting the oocyst cell wall and facilitating the release of DNA for its further extraction needs to be adapted. To improve the extraction yield, several pretreatment protocols have been proposed, based on thermal (cycles of freeze-thawing) [18], chemical (i.e., reducing agents, lytic enzymes), or mechanical (i.e., blade or pestle, ultrasonication, pressure cell, bead beating) disruption of the oocysts [19]. However, recent studies have reported the highest performances of the mechanical pretreatment using grinding beads for DNA extraction from *Cryptosporidium* sp. oocysts [9,10].

In this context, we recently evaluated six extraction protocols associated with various mechanical pretreatments for *C. parvum* oocyst DNA extraction in a multicenter comparative study [20]. This study highlighted the importance of sample pretreatment, as well as the extraction method, to improve the diagnostic performances of *C. parvum* DNA amplification methods. More precisely, it has been demonstrated that the automated extraction systems, using Boom technology associated with mechanical pretreatment using grinding beads, present the best performance for *Cryptosporidium* DNA extraction.

Nowadays, pretreatment protocols use mostly silica beads or glass beads for the grinding sample. However, no consensus of use according to bead type is clearly specified in the scientific literature or by manufacturers. Therefore, knowing that (i) the mechanical pretreatment has been proven to improve DNA extraction from *Cryptosporidium* oocysts [9,10], (ii) the speed and duration features of the grinding step influence extraction performances, and (iii) most manufacturers do not provide technical recommendations for the mechanical pretreatment to improve DNA extraction from *Cryptosporidium* sp., routine practices vary between laboratories, and discrepancies exist in the PCR detection performances of *Cryptosporidium* sp. DNA. Thus, in order to go further in *Cryptosporidium* sp. DNA extraction protocol optimization, the aim of this complementary study was to evaluate the impact of the physicochemical parameters of the grinding beads used in the mechanical pretreatment on *Cryptosporidium* sp. DNA extraction performances.

## 2. Materials and Methods

**Figure 1:**
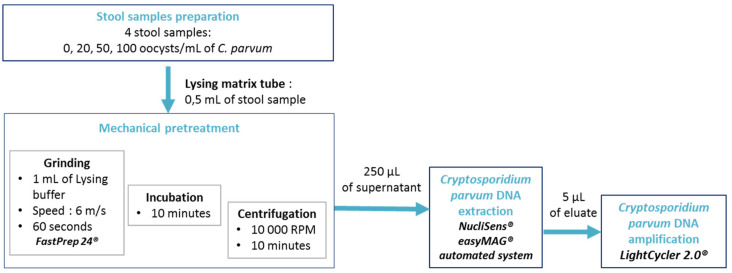
Experimental protocol flow chart (RPM: revolution per minute).

This study was conducted between 27 July to 5 September 2020 at the parasitology laboratory of the University Hospital of Dijon, which has proficiencies in the molecular detection of *Cryptosporidum* sp. DNA from stools. The step-by-step protocol is detailed in Figure 1.

### 2.1. Design of the Study

In order to study the impact of the physicochemical parameters of the grinding beads for *C. parvum* DNA extraction and DNA amplification, eleven mechanical lysis matrixes were tested. As a reminder, a mechanical lysis matrix is a matrix composed of beads and/or particles of variable sizes, shapes, and chemical compositions, used for mechanical pretreatment. All in all, four stool samples with concentrations of oocysts ranging from 0 to 100 oocysts/mL were tested per mechanical lysis matrix. The performances of the mechanical lysis matrixes were first evaluated by comparing the average percentage of positive *Cryptosporidium parvum* PCRs over the total number of PCRs performed at each *C. parvum* oocysts concentration for each mechanical lysis matrix, as described by Cha et al., 2014 [21].

### 2.2. Stool Samples Preparation

The CNR-LE for cryptosporidiosis (University Hospital of Rouen, Rouen, France) provided oocysts of *C. parvum* subtype IIaA15G2R1 from diarrheal stools of young calves. In order to evaluate the impact of the physicochemical parameters of the grinding beads for *C. parvum* DNA extraction and DNA amplification, four stool samples with various concentration of *C. parvum* sp. oocysts were prepared using human feces negative as a matrix (i.e., negative human feces for (i) common digestive parasites by microscopy and (ii) for *Cryptosporidium* sp., *Entamoeba* sp., *Giardia duodenalis*, *Enterocytozoon bieneusii*, and *Encaphalitozoon intestinalis* by PCR methods). Type 7 stools, according to the Bristol Stool Form Scale (BSFS), were prepared from this stool according to the following protocol: 20 g of stool in 50 mL of physiological saline (0.09% NaCl), filtered through a large mesh strainer and stored at 4 °C. The number of DNA extractions varied with the parasite concentration tested, and was higher for the lowest concentrations to fit Poisson’s law (a maximum of five extractions was carried out at the 10 oocysts/mL concentration) (Table 1). For each of the mechanical lysis matrixes tested, 10 stool samples containing 0 (*n* = 1), 20 (*n* = 5), 50 (*n* = 2), and 100 (*n* = 2) oocysts/mL were prepared. The stool samples were stored at 4 °C until the experiment.

### 2.3. Mechanical Pretreatment

The eleven mechanical lysis matrixes tested in this study were composed of beads with different sizes, shapes, and molecular compositions, which relied on two important parameters of each matrix: the hardness and the density (Table 2). These eleven mechanical lysis matrixes can be divided into three distinct groups according to their chemical composition: (a) glass or silica, (b) garnet, and (c) technical ceramic (i.e., zirconium stabilized with cerium oxide, aluminum oxide, silicon carbide, yellow zirconium, and zirconium silicate). Mechanical grinding was carried out by applying the following protocol: 0.5 mL of stool sample was added to each of the mechanical lysis matrixes with 1 mL of NucliSenS^®^ lysing buffer before being grounded using the FastPrep 24^®^ grinder/homogenizer (MP Biomedical, Irvine, CA, USA) at a speed of 6.0 m/s for 60 s, offering the best performances for *Cryptosporidium* sp. DNA, as reported by Valeix et al. [20].

### 2.4. Cryptosporidium parvum DNA Extraction

*Cryptosporidium parvum* DNA was extracted with the NucliSENS^®^ easyMAG^®^ automated system (BioMérieux, Marcy-l’Etoile, France) following the protocol by Jeddi et al., 2013 [10]. Briefly, after the mechanical grinding step, the stool suspension obtained was then incubated at room temperature for 10 min before being centrifuged at 10,000 g for 10 min. Finally, 250 μL of supernatant was transferred in the DNA extraction NucliSENS^®^ easyMAG^®^ automated system (BioMérieux, Marcy-l’Etoile, France) with 50 µL of NucliSENS^®^ EasyMAG^®^ magnetic silica (Biomérieux, Marcy-l’Etoile, France). Elution was performed at RT with 100 μL of elution buffer. The eluted DNA volume obtained (100 μL) was then stored at 4 °C. PCR amplification was then performed within 10 days after DNA extraction.

### 2.5. Cryptosporidium parvum DNA Amplification

For *Cryptosporidium parvum* DNA amplification, our in-house PCR was used following the protocol as described in Brunet et al., 2016 [22]. Briefly, the amplification of a 258-bp DNA fragment located in the 18S ribosomal ribonucleic acid (rRNA) gene (GenBank accession n°L16996; positions 80 to 337) was carried out using the forward 5′ GTT AAA CTG CRA ATG GCT 3′ (Cry80F3) and reverse 5′ CGT CAT TGC CAC GGT A 3′ (Cry337R) primers, using the hybridization probes: 5 ′CCG TCT AAA GCT GAT AGG TCA GAA ACT TGA ATG 3′ Fluorescein (anchor probe) and 5′ Red 640-GTC ACA TTA ATT GTG ATC CGT AAA G 34 Phosphate (sensor probe). Primers and probes were used at a concentration of 10 μM. Five microliters of DNA extracts were added to a final reaction volume of 20 μL to each amplification reaction tube. Thermocycling and fluorescence detection were performed on the LightCycler 2.0 Roche Molecular Systems, Inc. (Rotkreuz, Switzerland). One negative (i.e., sterile water or stool samples without *Cryptosporidium* oocysts and other parasites) and one positive (i.e., stool samples containing *C. parvum* at the concentration of 100 oocysts/mL) controls were included in each assay. All in all, a total of 625 PCRs were carried out, including 605 *Cryptosporidium*-specific PCRs for *Cryptosporidium* DNA detection in stool extracts, and 20 PCRs for the internal control detection in stool extracts.

### 2.6. Statistical Analysis

The statistical analyses were performed using the BioStaTGV and GraphPad PRISM softwares. The PCR detection percentages with the different mechanical lysis matrixes were compared using the chi-square test. In case of small sample sizes, Fisher’s exact test was used. The Ct values found by PCR were compared among the different mechanical lysis matrixes using the Kruskal–Wallis test, followed when appropriate (omnibus *p*-value less than 0.05) by Dunn’s two-by-two post hoc test. A probability of 0.05 or less was considered to be significant.

## 3. Results

### 3.1. Influence of Mechanical Lysis Matrixes for Cryptosporidium parvum DNA Amplification

All of the negative controls included in the study were negative by PCR. For the positive samples, the performances in *C. parvum* DNA amplification were variable depending on the mechanical lysis matrix tested. Globally, Lysing Matrix D^®^ showed the best performances, with an average positive rate of 94.4%. Oppositely, Lysing Matrix B^®^ and Lysing Matrix C^®^ had the lowest performances, with average positive rates of 64.8 and 53.7%, respectively. The other mechanical lysis matrixes included in this study displayed acceptable performances, with average positivity rates ranging from 79.6% to 90.7% (Table 3).

When analyzing the results depending on the *C. parvum* oocyst concentration, significant differences were observed at the concentration of 20 oocysts/mL between the eleven mechanical lysis matrixes studied, with the positive rate varying from 26.7 to 90.0% (Table 3 and Table 4). More precisely, at the concentration of 20 oocysts/mL, Lysing Matrix B^®^ and Lysing Matrix C^®^ showed the lowest performances, with positive rates of 40.0 and 26.7%, respectively. The other nine Lysing Matrix (i.e., A^®^, D^®^, E^®^, F^®^, G^®^, H^®^, I^®^, J^®^, and K^®^) showed comparable results for this concentration, with positive rate varying from 63.3% to 90% (Table 3 and Table S1). Finally, the eleven mechanical lysis matrixes showed no significant differences at concentrations of 50 and 100 oocysts/mL (Table 3).

### 3.2. Influence of Mechanical Lysis Matrixes on the Average Cycle Threshold (Ct) Values Obtained during Cryptosporidium parvum DNA Amplification by PCR

The average cycle threshold (Ct) obtained for the external control was 33 Ct, with a standard deviation of 0.80. For the spiked samples, the specificity was confirmed with the melting curve (i.e., melting temperature (Tm) of 53.5 °C for *C. parvum*). All in all, Lysing Matrix D^®^ and Lysing Matrix E^®^ showed the best performances, with mean Ct values ranging from 33.58 ± 0.45 to 37.47 ± 3.20, and from 33.71 ± 0.62 to 38.05 ± 4.16, respectively. Oppositely, Lysing Matrix B^®^ and Lysing Matrix C^®^ showed the lowest performances, with mean Ct values ranging from 35.23 ± 1.34 to 42.25 ± 3.81, and from 35.67 ± 2.54 to 43.53 ± 2.85, respectively (Table 4).

**Figure 2:**
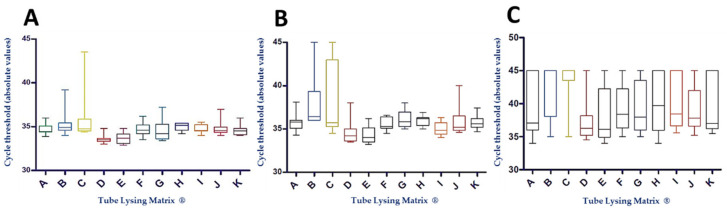
Distribution of the Ct results obtained according to the mechanical lysing matrix at the concentration of (**A**) 100 oocysts/mL, (**B**) 50 oocysts/mL, and (**C**) 20 oocysts/mL.

At the concentration of 100 oocysts/mL, two mechanical lysing matrixes stood out from the others: (i) Lysing Matrix D^®^, which displayed Ct values statistically lower than those obtained with Lysing Matrixes B^®^, C^®^, F^®^, H^®^, I^®^, and J^®^, and which also appeared to be more reproducible, with a lower dispersion of the Ct, and (ii) Lysing Matrix E^®^, which showed Ct values statistically lower than those obtained with Lysing Matrixes B^®^, C^®^, and H^®^ (Figure 2A and Table S2).

At the concentration of 50 oocysts/mL, Lysing Matrix B^®^ exhibited the worst performances, with Ct values significantly higher than those obtained with Lysing Matrixes D^®^, E^®^, and I^®^, while Lysing Matrix E^®^ showed the best results, with Ct values significantly lower than those obtained with Lysing Matrixes B^®^, C^®^, G^®^, and H^®^. Furthermore, Lysing Matrix C^®^ showed a greater dispersion of results than the others (Figure 2B and Table S3).

At the lower concentration of 20 oocysts/mL, Lysing Matrix D^®^ and Lysing Matrix E^®^ showed higher rates of DNA extraction and amplification, linked to lower Ct values. A significant difference was observed between the Ct values for Lysing Matrix D^®^ and Lysing Matrix E^®^ and those of Lysing Matrix B^®^ and Lysing Matrix C^®^. Finally, Lysing Matrix C^®^ showed poorer performances, with Ct values significantly higher than those of Lysing Matrixes A^®^, D^®^, E^®^, F^®^, G^®^, J^®^, and K^®^ (Figure 2C and Table S4).

## 4. Discussion

Recently, our team highlighted the importance of sample pretreatment, as well as the extraction method, on the diagnostic performances of the *C. parvum* DNA amplification methods [20]. In this context, and in order to go further in the improvement of the *C. parvum* DNA amplification methods, we focused this complementary study on the evaluation of the impact of the mechanical lysing matrix used for the pretreatment of stool samples for PCR detection of *Cryptosporidium* DNA. The eleven Lysis Matrixes^®^ (MP Biomedical^®^) included in this study showed variable performances, dispatched into three distinct groups: (1) including Lysis Matrixes B^®^ and C^®^, which had the lowest performances for *C. parvum* DNA extraction and amplification, (2) gathering Lysis Matrixes A^®^, F^®^, G^®^, H^®^, I^®^, J^®^, and K^®^, which showed comparable intermediate efficiency, and (3) including Lysis Matrixes E^®^ and D^®^, which achieved the best performances for *C. parvum* DNA amplification. It is interesting to note that the only matrix indicated (Lysing Matrix B^®^) by the manufacturer for oocysts pretreatment is one of the matrixes with the poorest performance, hence the importance of this type of study.

**Figure 3:**
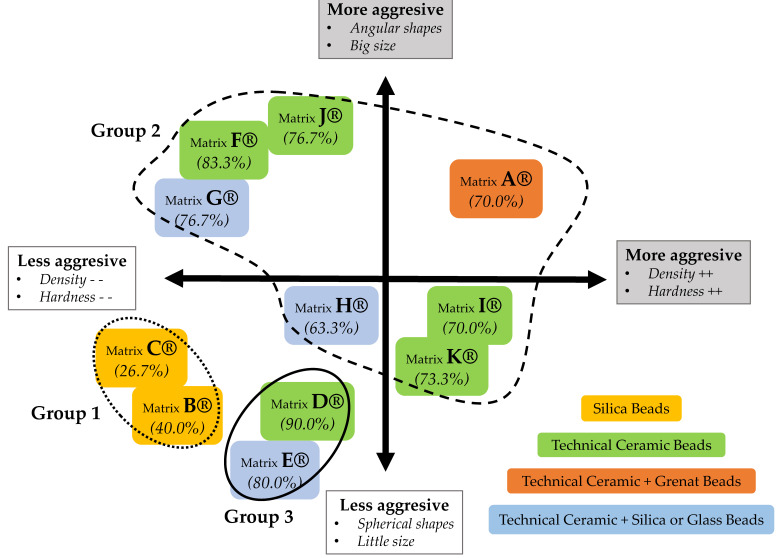
Repartition of the eleven mechanical lysing matrixes according to their characteristics and performances (percentage of detection at the concentration of 20 oocysts/mL).

Thus, our data corroborate the view that the characteristics of the beads used in lysis matrixes, including bead size, shape, and molecular composition, influence the performances of the pretreatment step (Figure 3).

Globally, the results obtained for the three groups of components tested during this study are consistent with those observed in our previous study [20]. Thus, the technical ceramic matrixes were the most efficient, particularly when used alone. Conversely, the glass or silica beads presented the worse performances used alone, and also showed a negative impact in combination with other components by reducing the overall performances. Moreover, we highlighted that shape and chemical composition are closely linked. Therefore, the same chemical nature and spherical shape, leading to mechanical lysis by crushing forces, seems to present better performance for *C. parvum* oocysts DNA extraction than angular shape, carrying out lysis by shear forces. Thus, aggressive pretreatment could result in a decrease in the overall extraction performances. Indeed, mechanical pretreatment could induce either a *Cryptosporidium* sp. DNA alteration or simply doesn’t allow expulsion of *Cryptosporidium* sp. DNA from the oocyst. Furthermore, as previously reported, the use of smaller beads seems to be efficient for the lysis of small structures, such as oocysts. Indeed, in the case of the extraction and excystation of oocysts of *Eimeria* sp., it has been shown that the use of small glass beads (0.5 mm) effectively broke the wall of oocysts, while larger ones preserved sporozoites [21,23].

All in all, the molecular composition, shape, and size of the particles of the lysis matrix influence the extraction performance by determining two essential qualities for the grinding step: hardness and density. Indeed, the constituent elements of the lysis matrix need to combine (i) a hardness greater than that of the wall of the oocyst, and (ii) a density close to the buffer used during the pretreatment, so that the beads do not float, aimed at efficiently disrupting oocysts for *Cryptosporidium* sp. DNA extraction [11,21,23]. However, as demonstrated in our previous study, the use of glass beads for the pretreatment step will result in variable performances, depending on the extraction protocol used [20]. Thus, it is important to consider the pretreatment step in association with the extraction method.

## 5. Conclusions

Nowadays, many commercial kits allow the detection of *Cryptosporidium* sp. DNA in stool specimens, but the complex physicochemical features of the *Cryptosporidium* sp. oocyst wall require optimization and standardization of the extraction protocols. We previously highlighted the importance of sample pretreatment, as well as the extraction method, to improve the diagnostic performances of the *C. parvum* DNA amplification methods. Here, in this complementary study, we showed the importance of beads’ physicochemical characteristics on the success of the pretreatment step, particularly the shape and chemical composition, which are closely related.

## Figures and Tables

**Table 1:** Design of the study: the number of DNA extractions and *Cryptosporidium*-specific PCRs according to the parasite concentration for each of the grinding matrixes tested.

Stool Concentration (Oocysts/mL)	No. of Extractions Done per Method	No. of *Cryptosporidium*-Specific PCRs	
		per Extraction	Total
0	1	1	1
10	5	6	30
50	2	6	12
100	2	6	12
All	10	-	55

**Table 2:** The composition and properties of the mechanical lysis matrixes according to data from the manufacturer (MP Biomedical^®^) HV: hardness according to the Vickers scale.

Commercial Denomination (MP Biomedical^®^)	Mechanical Lysis Matrix				Relative Density	Hardness (Mohs Scale)	Manufacturer Recommendations for Sample Types Grindable According Lysis Matrix
	Number of Elements	Size (Diameter in mm)	Shape	Chemical Composition (Molecular Formula)			
Lysing Matrix **A**^®^	1	6.35	Bead with a satellite band	Zirconium sphere with banded satellite (ZrO_2_ 88%, CeO 10%)	5.5–6.1	6–7 (1050 HV)	**Animals**: tissues and cells (heart, lungs, hair, teeth, tumors, bones, etc.) **Microbiology**: fungi, yeasts, bacteria (gram-positive and negative) **Environment**: plants, soils, wastewater, sludge, feces
	Many	0.56–0.7	Flakes	Garnet (Fe_3_Al_2_(SiO_4_)_3_)	4.0–4.1	7.5–8	
Lysing Matrix **B**^®^	Many	0.1	Beads	Silica (SiO_2_)	2.5	5–6	**Microbiology and environment**: spores, bacteria (gram-positive and negative), archaea, oocysts, prokaryotes
Lysing Matrix **C**^®^	Many	1	Beads	Silica (SiO_2_)	2.5	5–6	**Microbiology and environment**: spores, fungi, yeasts, algae
Lysing Matrix **D**^®^	Many	1.4	Beads	Ceramic	4	5–6 (800 HV)	**Animals**: soft tissues (kidneys, liver, brain, spleen), cell cultures **Environment**: plants (fruits and roots), whole insects
Lysing Matrix **E**^®^	Many	1.4	Beads	Ceramic	4	5–6 (800 HV)	**Animals**: microbe infected tissue, tumors and other difficult tissues **Environment**: feces, soil, wastewater and environmental water, sludge, pollen
	Many	0.1	Beads	Silica (SiO_2_)	2.5	5–6	
	1	4	Bead	Glass (SiO_2_)	2.5	5–6	
Lysing Matrix **F**^®^	Many	1.6	Particles	Aluminum oxide (Al_2_O_3_)	3.95	9	**Animals**: tissues **Microbiology**: bacteria (gram-positive and negative), fungi **Environment**: plants, coral emulsions, mold
	Many	1.2–2.4	Particles	Silicon carbide (SiC)	3.20	9–10	
Lysing Matrix **G**^®^	Many	1.2–2.4	Particles	Silicon carbide (SiC)	3.20	9–10	**Animals**: tissues **Microbiology**: fungi, spores, yeasts
	Many	1.7–2.1	Beads	Glass (SiO_2_)	2.5	5–6	
Lysing Matrix **H**^®^	Many	1.7–2.1	Beads	Glass (SiO_2_)	2.5	5–6	**Animals**: tissues **Environment**: whole insects, plants, soils, clays, wood, dried samples **Microbiology**: bacterial aggregates and biofilms
	Many	1.7–2.3	Beads	Yellow zirconium oxide (ZrO_2_ 97%, MgO 3%)	5.5	6–7 (900–1000 HV)	
Lysing Matrix **I**^®^	Many	1.7–2.3	Beads	Yellow zirconium oxide (ZrO_2_ 97%, MgO 3%)	5.5	6–7 (900–1000 HV)	**Animals**: tissues, exoskeleton of arthropods and crustaceans **Microbiology**: spores **Environment**: plants, dense soils and clays, wood
	Many	3.7–4.2	Beads	Ceramic	6.0–6.25	7,5	
Lysing Matrix **J**^®^	Many	1.7–2.3	Beads	Yellow zirconium oxide (ZrO_2_ 97%, MgO 3%)	5.5	6–7 (900–1000 HV)	**Animals**: tissues **Microbiology**: bacteria gram-positive and negative), spores, cysts, fungi, molds **Environment**: plants, coral emulsions, mold
	Many	1.6	Beads	Aluminum oxide (Al_2_O_3_)	3.95	9	
Lysing Matrix **K**^®^	Many	0.8	Beads	Zirconium silicate (ZrO_2_ 68%, SiO_2_ 32%)	4	7.0	**Animals**: tissues, bones. **Microbiology**: fungal spores, cysts, yeast polysaccharide capsules, fixed, old or dried samples.

**Table 3:** Performances of the eleven mechanical lysis matrixes studied for *Cryptosporidium parvum* DNA extraction and amplification.

Lysing Matrix^®^		A	B	C	D	E	F	G	H	I	J	K
**Overall Proportion of Positive Samples (%)**		83.3	64.8	53.7	94.4	88.9	90.7	87.0	79.6	83.3	87.0	85.2
**Proportion of Positive Samples at Each Concentration (%)**	20 oocysts/mL	70.0	40.0	26.7	90.0	80.0	83.3	76.7	63.6	70.0	76.7	73.3
	50 oocysts/mL	100	91.7	75.0	100	100	100	100	100	100	100	100
	100 oocysts/mL	100	100	100	100	100	100	100	100	100	100	100

**Table 4:** Average of Ct values and standard deviations of PCR results according to the mechanical lysing matrix and oocyst concentration.

		Lysing Matrix^®^										
		A	B	C	D	E	F	G	H	I	J	K
**Average of Ct Values ± Standard Deviation**	100 oocysts/mL	34.68 ± 0.57	35.23 ± 1.34	35.67 ± 2.54	33.58 ± 0.45	33.71 ± 0.62	34.74 ± 0.77	34.55 ± 1.23	35.03 ± 0.43	34.72 ± 0.45	34.78 ± 0.78	34.62 ± 0.57
	50 oocysts/mL	35.84 ± 1.18	38.07 ± 3.04	37.91 ± 4.31	34.74 ± 1.42	34.38 ± 1.03	35.59 ± 0.75	36.13 ± 1.01	35.94 ± 0.59	35.06 ± 0.74	35.85 ± 1.52	35.75 ± 0.71
	20 oocysts/mL	39.12 ± 4.15	42.25 ± 3.81	43.53 ± 2.85	37.47 ± 3.20	38.05 ± 4.16	39.27 ± 3.51	39.40 ± 3.71	40.15 ± 4.14	40.11 ± 3.76	39.22 ± 3.52	39.28 ± 3.84

## Supplementary Materials

The following are available online at https://www.mdpi.com/2076-2607/9/2/297/s1, Table S1: Degrees of statistical significance of Chi2 tests comparing the percentage of positive *Cryptosporidium parvum* PCR according to the mechanical lysis matrix used for the concentrations of 20 oocysts/mL; Table S2: Degrees of statistical significance of Mann-Whitney tests comparing mean Ct values of *Cryptosporidium parvum* PCRs according to mechanical lysing matrix for the concentrations of 100 oocysts/mL., Table S3: Degrees of statistical significance of Mann-Whitney tests comparing mean Ct values of *Cryptosporidium parvum* PCRs according to mechanical lysing matrix for the concentration of 50 oocysts/mL., Table S4: Degrees of statistical significance of Mann-Whitney tests comparing mean Ct values of *Cryptosporidium parvum* PCR according to mechanical lysing matrix for the concentrations of 20 oocysts/mL
