# Supplementary material for: Comparative Study of Eleven Mechanical Pretreatment Protocols for Cryptosporidium parvum DNA Extraction from Stool Samples
Source: Microorganisms. 2021 Feb 2;9(2):297. doi: 10.3390/microorganisms9020297 (PMC7912823; doi:10.3390/microorganisms9020297)
Supplement: Supplementary file 1 [file microorganisms-09-00297-s001.pdf]

SUPPLEMENTAL DATA

Table S1. Degrees of statistical significance of Chi2 tests comparing the percentage of positive *Cryptosporidium parvum* PCR according to the mechanical lysis matrix used for the concentrations of 20 oocysts/mL.

Tube Lysing Matrix ®	A	B	C	D	E	F	G	H	I	J	K
A		*	***	ns	ns	ns	ns	ns	ns	ns	ns
B	*		ns	***	**	***	**	ns	*	**	**
C	***	ns		***	***	***	***	**	***	***	***
D	ns	***	***		ns	ns	ns	*	ns	ns	ns
E	ns	**	***	ns		ns	ns	ns	ns	ns	ns
F	ns	***	***	ns	ns		ns	ns	ns	ns	ns
G	ns	**	***	ns	ns	ns		ns	ns	ns	ns
H	ns	ns	**	*	ns	ns	ns		ns	ns	ns
I	ns	*	***	ns	ns	ns	ns	ns		ns	ns
J	ns	**	***	ns	ns	ns	ns	ns	ns		ns
K	ns	**	***	ns	ns	ns	ns	ns	ns	ns	

** ($p \leq 0.05$), ** ($p \leq 0.01$), *** ($p \leq 0.001$), ns: not significant.*

Table S2. Degrees of statistical significance of Mann-Whitney tests comparing mean Ct values of *Cryptosporidium parvum* PCRs according to mechanical lysing matrix for the concentrations of 100 oocysts/mL.

Tube Lysing Matrix®	A	B	C	D	E	F	G	H	I	J	K
A		ns	ns	ns	ns	ns	ns	ns	ns	ns	ns
B	ns		ns	***	**	ns	ns	ns	ns	ns	ns
C	ns	ns		***	**	ns	ns	ns	ns	ns	ns
D	ns	***	***		ns	*	ns	***	*	*	ns
E	ns	**	**	ns		ns	ns	***	ns	ns	ns
F	ns	ns	ns	*	ns		ns	ns	ns	ns	ns
G	ns	ns	ns	ns	ns	ns		ns	ns	ns	ns
H	ns	ns	ns	***	***	ns	ns		ns	ns	ns
I	ns	ns	ns	*	ns	ns	ns	ns		ns	ns
J	ns	ns	ns	*	ns	ns	ns	ns	ns		ns
K	ns	ns	ns	ns	ns	ns	ns	ns	ns	ns	

** ($p \leq 0.05$), ** ($p \leq 0.01$), *** ($p \leq 0.001$), ns: not significant. (n=12 Ct values)*

Table S3. Degrees of statistical significance of Mann-Whitney tests comparing mean Ct values of *Cryptosporidium parvum* PCRs according to mechanical lysing matrix for the concentration of 50 oocysts/mL.

Tube Lysing Matrix®	A	B	C	D	E	F	G	H	I	J	K
A		ns	ns	ns	ns	ns	ns	ns	ns	ns	ns
B	ns		ns	***	***	ns	ns	ns	**	ns	ns
C	ns	ns		ns	*	ns	ns	ns	ns	ns	ns
D	ns	***	ns		ns	ns	ns	ns	ns	ns	ns
E	ns	***	*	ns		ns	*	*	ns	ns	ns
F	ns	ns	ns	ns	ns		ns	ns	ns	ns	ns
G	ns	ns	ns	ns	*	ns		ns	ns	ns	ns
H	ns	ns	ns	*	*	ns	ns		ns	ns	ns
I	ns	**	ns	ns	ns	ns	ns	ns		ns	ns
J	ns	ns	ns	ns	ns	ns	ns	ns	ns		ns
K	ns	ns	ns	ns	ns	ns	ns	ns	ns	ns	

** ($p \leq 0.05$), ** ($p \leq 0.01$), *** ($p \leq 0.001$), ns: not significant. (9<= n<=12 Ct values)*

Table S4. Degrees of statistical significance of Mann-Whitney tests comparing mean Ct values of *Cryptosporidium parvum* PCR according to mechanical lysing matrix for the concentrations of 20 oocysts/mL.

Tube Lysing Matrix®	A	B	C	D	E	F	G	H	I	J	K
**A**		ns	**	ns	ns	ns	ns	ns	ns	ns	ns
**B**	ns		ns	***	***	ns	ns	ns	ns	ns	ns
**C**	**	ns		***	***	**	*	ns	ns	*	**
**D**	ns	***	***		ns	ns	ns	ns	ns	ns	ns
**E**	ns	***	***	ns		ns	ns	ns	ns	ns	ns
**F**	ns	ns	**	ns	ns		ns	ns	ns	ns	ns
**G**	ns	ns	*	ns	ns	ns		ns	ns	ns	ns
**H**	ns	ns	ns	ns	ns	ns	ns		ns	ns	ns
**I**	ns	ns	ns	ns	ns	ns	ns	ns		ns	ns
**J**	ns	ns	*	ns	ns	ns	ns	ns	ns		ns
**K**	ns	ns	**	ns	ns	ns	ns	ns	ns	ns	

** ($p \leq 0.05$), ** ($p \leq 0.01$), *** ($p \leq 0.001$), ns: not significant. ($n \geq 8$ Ct values)*
